# Perspective of Information Technology Decision Makers on Factors Influencing Adoption and Implementation of Artificial Intelligence Technologies in 40 German Hospitals: Descriptive Analysis

**DOI:** 10.2196/34678

**Published:** 2022-06-15

**Authors:** Lina Weinert, Julia Müller, Laura Svensson, Oliver Heinze

**Affiliations:** 1 Institute of Medical Informatics Heidelberg University Hospital Heidelberg Germany

**Keywords:** artificial intelligence, AI readiness, implementation, decision-making, descriptive analysis, quantitative study

## Abstract

**Background:**

New artificial intelligence (AI) tools are being developed at a high speed. However, strategies and practical experiences surrounding the adoption and implementation of AI in health care are lacking. This is likely because of the high implementation complexity of AI, legacy IT infrastructure, and unclear business cases, thus complicating AI adoption. Research has recently started to identify the factors influencing AI readiness of organizations.

**Objective:**

This study aimed to investigate the factors influencing AI readiness as well as possible barriers to AI adoption and implementation in German hospitals. We also assessed the status quo regarding the dissemination of AI tools in hospitals. We focused on IT decision makers, a seldom studied but highly relevant group.

**Methods:**

We created a web-based survey based on recent AI readiness and implementation literature. Participants were identified through a publicly accessible database and contacted via email or invitational leaflets sent by mail, in some cases accompanied by a telephonic prenotification. The survey responses were analyzed using descriptive statistics.

**Results:**

We contacted 609 possible participants, and our database recorded 40 completed surveys. Most participants agreed or rather agreed with the statement that AI would be relevant in the future, both in Germany (37/40, 93%) and in their own hospital (36/40, 90%). Participants were asked whether their hospitals used or planned to use AI technologies. Of the 40 participants, 26 (65%) answered “yes.” Most AI technologies were used or planned for patient care, followed by biomedical research, administration, and logistics and central purchasing. The most important barriers to AI were lack of resources (staff, knowledge, and financial). Relevant possible opportunities for using AI were increase in efficiency owing to time-saving effects, competitive advantages, and increase in quality of care. Most AI tools in use or in planning have been developed with external partners.

**Conclusions:**

Few tools have been implemented in routine care, and many hospitals do not use or plan to use AI in the future. This can likely be explained by missing or unclear business cases or the need for a modern IT infrastructure to integrate AI tools in a usable manner. These shortcomings complicate decision-making and resource attribution. As most AI technologies already in use were developed in cooperation with external partners, these relationships should be fostered. IT decision makers should assess their hospitals’ readiness for AI individually with a focus on resources. Further research should continue to monitor the dissemination of AI tools and readiness factors to determine whether improvements can be made over time. This monitoring is especially important with regard to government-supported investments in AI technologies that could alleviate financial burdens. Qualitative studies with hospital IT decision makers should be conducted to further explore the reasons for slow AI.

## Introduction

### Background

In recent years, artificial intelligence (AI) in medicine has gained significant attention, with innovative technologies promising better quality of diagnosis [1-3], treatment [1], advancements in personalized medicine [1,4], and improvements in workflow [5]. Simultaneously, these technologies have the potential to save time and cost [1,6]. The use of AI could free health care workers from repetitive and tedious tasks and enable them to allocate their attention and time more effectively [7]. However, fears surrounding AI in health care persist. Common fears include possible job losses because of automation and negative effects on the patient-physician relationship [2,8,9]. For this study, we used the definition by He et al [10]. They define AI as *“*a branch of applied computer science wherein computer algorithms are trained to perform tasks typically associated with human intelligence*”* [10]*.* There are different relevant subcategories of AI, such as machine learning and deep learning, with different implications for professional users and health care organizations. However, in this study, we focused on the general concept of AI in hospitals.

A recent systematic review by Yin et al [5] demonstrated hesitancy and slow uptake of AI technologies. The authors reported on real-life implementations of AI in health care. Their search retrieved 51 real-life clinical implementations of AI worldwide, with most studies conducted in the United States. The most common applications of AI tools were in the field of decision support. These technologies mainly focus on specific diseases such as sepsis, breast cancer, and diabetic retinopathy [5]. Diverging outcome measures and low-quality studies were prevalent in the review, making it difficult for decision makers to compare and evaluate AI effectiveness, advantages, and disadvantages. Furthermore, they found that outcome evaluation and acceptance measures only included patients and health care workers [5]. Their search strategy retrieved only one paper from Germany, which is in contrast with the German government’s AI strategy [11] and recent political efforts to increase the use of AI in hospitals [12]. Hence, we identified a need to investigate the current spread of AI technologies in hospitals and their stage of development as well as AI readiness factors in Germany.

The transfer of new and innovative technologies into practice is usually associated with barriers and requires employees’ and institutions’ ability to adapt to change [13,14]. Recently, existing frameworks and learnings on the dissemination of innovative technologies have been applied to AI [15]. Three main components can be outlined: (1) adoption, which entails the decision to use an innovation [16]; (2) readiness, encompassing the assessment of the conditions needed to engage in an activity [17]; and (3) implementation, describing an innovation’s transfer into practice [15].

Although new AI technologies are being developed at a high speed, strategies and practical experiences surrounding the adoption and implementation of AI in health care are lacking [10,18]. This is partly because of the high implementation complexity of AI, as it is neither easy to use nor easy to deploy [17,19]. Furthermore, AI can be difficult to understand and has been described as a *black box*, meaning a machine with nontransparent workings and inexplicable results of automated algorithms. This has the potential to lower trust and discourage decision makers and users [4,20,21].

### Aims

This study presents the first large-scale web-based survey on the current adoption and implementation of AI technologies in randomly selected German hospitals. We further aimed to gain insights into the number, type, and developmental stage of the AI technologies currently in use. In addition to the literature on AI readiness and adoption, we examined the applicability of existing AI readiness factors to the German health care sector.

## Methods

### Study Design

A quantitative study design was used to obtain a general overview of the situation in Germany. Data were collected using an anonymous web-based questionnaire. We invited chief information officers (CIOs) from randomly selected German hospitals. We identified CIOs as important intermediaries because their position is linked to the clinical implementation of AI as well as to developers, technology companies, and regulatory authorities. Anonymity was ensured throughout the study.

### Ethics Approval

The study was approved by the Ethics Committee of Heidelberg University Hospital (S-490/2020). The study was conducted according to the Checklist for Reporting Results of Internet E-Surveys checklist for quantitative research [22].

### Instrument Development and Design

After consulting existing literature on AI readiness, implementation, and adoption, the authors conducted a creative brainstorming process to develop preliminary survey items. The preliminary items were compared with existing theoretical frameworks.

Jöhnk et al [15] developed a model that focused on organizational AI readiness. They described AI readiness both as a predecessor and a constant influence on AI adoption and implementation [15]. Jöhnk et al [15] identified 18 organizational readiness factors in 5 categories (strategic alignment, resources, knowledge, culture, and data) and pointed out that these factors continuously foster AI adoption [15]. Awareness of these factors can improve the adoption and implementation outcomes, as a higher level of organizational readiness is believed to increase the success of innovation adoption while lowering the risk of failure [20,23]. For example, knowledge and awareness of AI were shown to be prerequisites for successful AI adoption [15,24,25].

The technological-organizational-environmental framework by DePietro et al [26] describes the adoption, implementation, and use of technology in firms as dependent on the technological, organizational, and the environmental context [27]. Pumplun et al [24] first applied this framework to AI and discussed that challenges to AI readiness can be observed at all of these levels. Observed technological challenges often stem from data accessibility issues owing to AI’s need for extensive databases and adjacent data privacy considerations [24,28]. Environmental challenges include questions about consumer and patient trust in AI, regulatory acceptance, and in some cases, mandated work councils (mandated institutions of nonunion employee representation) [6,24,29,30]. Concerning organizational challenges, a lack of (top) managerial support has been identified as very relevant [17]. A further challenge is the need for highly skilled and trained staff (eg, data scientists, a very sought-after group of professionals) [15,17]. Financial aspects, such as unclear reimbursement processes for health care delivered by AI and liability issues, contribute to hesitancy in AI adoption and implementation [1].

On the basis of these theoretical considerations, LW, JM, and LS refined the survey design and wording of the questions. In the first section, the questionnaire focused on participants’ general professional opinions on AI in hospitals to assess the hospital’s strategic alignment and their stance in the AI adoption phase. The second section asked participants to state their hospital’s use of AI technologies, which helped us gain insight into the dissemination of AI technologies. In the following sections, the survey presents items on known perceived barriers, opportunities, and resources needed for the implementation of AI in hospitals. In addition to these questions, the questionnaire also asked for sociodemographic data of the participants, hospital size, and hospital ownership (private, public, or nonprofit). A translated English version of the survey can be found in Multimedia Appendix 1.

The survey was pretested by 6 researchers from the field of medical informatics, using a cognitive pretesting method [31]. The pretest participants suggested changes in the wording and order of questions. These suggestions were implemented, and the final survey was created.

The final survey did not include any randomized or alternated items. Adaptive questioning was used to reduce the length of the questionnaires. On average, the 10-page questionnaire contained 6.3 items per page. Possible answers were either presented on a 5-point Likert scale or as *yes or no*, with *I don’t know* and *prefer not to say* as alternative options. Few questions were asked for further elaboration of answers in open-text formats. Automatic checks for completeness were performed, and participants were required to choose an answer for each question. Cookies were used to assign unique user IDs. Participants were offered the option to return and modify their answers. They were also able to leave the survey and continue it later. IP addresses of participants were neither saved nor checked. REDCap (Research Electronic Data Capture; Vanderbilt University) [32,33] hosted at the Heidelberg University Hospital was used for data collection and management. REDCap is a secure web-based software platform designed to support data capture for research studies [32,33].

### Data Collection and Analysis

From a publicly available database of all hospitals in Germany provided by the German Hospital Federation [34], we randomly selected the hospitals we wanted to include in our recruitment process by performing a spreadsheet calculation. We aimed for an equal, realistic representation of hospital size (measured through the number of hospital beds) in each sample. We then checked whether the selected hospitals were actually in operation. Other specific inclusion and exclusion criteria were not applied, as we wanted to depict a realistic reflection of all the hospitals in Germany. In addition to this random selection, we included all academic hospitals in Germany in our recruitment efforts. CIOs and their contact details were manually retrieved from the websites of hospitals. We recruited participants from 609 hospitals in 4 rounds of recruitment. Initially, participants were invited via email to participate in the study. The emails contained a link to access the open survey and information about the study (eg, purpose of the study, length of questionnaire, data protection guidelines, and investigators). As participation in this study was voluntary and anonymous, we regarded survey completion as consent for study participation and data use.

Although all 4 rounds followed the same administrative process, we used additional measures in recruitment rounds 3 and 4 to increase the number of participants. In round 3, we used telephonic prenotifications when an office telephone number was publicly available. In round 4 of recruitment, we designed invitational leaflets that were sent via mail. The leaflets encompassed a short informational text and a QR code, leading to the open survey. For each round, we sent 2 reminders via email. Our survey was not advertised elsewhere, as we wanted to include only members of our specific target group in the sample. No incentives were offered to the study participants.

Data were collected from October 2020 to February 2021. After completion, all data were exported from REDCap to SPSS statistical software (version 27, IBM). All data were checked for plausibility and analyzed by LW. Descriptive analyses were conducted. For open-item responses, recurring keywords and phrases were paraphrased and summarized.

## Results

### Overview

Our database recorded 50 surveys, of which 10 were terminated early, usually in the first third of the survey. A total of 40 surveys were fully completed and were included in the analysis, resulting in a response rate of 6.6%. Timeframes were analyzed, but no unusual timeframes were observed. No statistical corrections were performed.

### Demographic Characteristics

A total of 40 fully completed surveys were included in the analysis. Table 1 provides information on participant characteristics. Most participants were aged between 46 and 55 years (23/40, 58%), and most of the participants were male (33/40, 83%). Of the 40 participants, 26 (65%) said they were CIOs or leaders of the IT department of their institution. Other commonly mentioned professions included IT department employee (7/40, 18%) and research associate (4/40, 1%). Participants stated the ownership of their hospitals as follows: public hospital (30/40, 75%), nonprofit hospital (8/40, 20%), private hospital (2/40, 5%), and hospital with an academic affiliation (15/40, 38%)

**Table 1:** Participant characteristics (N=40).

Characteristics		Participants
**Gender, n (%)**		
	Female	5 (13)
	Male	33 (83)
	Prefer not to say	2 (5)
**Age group (years), n (%)**		
	26 to 35	2 (5)
	35 to 45	8 (20)
	46 to 55	23 (58)
	56 to 65	5 (13)
	>65	2 (5)
**Hospital ownership, n (%)**		
	Public	30 (75)
	Nonprofit	8 (20)
	Private	2 (5)
**Academic affiliation, n (%)**		
	Academic	15 (38)
	Nonacademic	25 (63)
**Number of beds in hospital, n (%)**		
	1 to 199	3 (8)
	200 to 399	5 (13)
	400 to 599	7 (18)
	600 to 799	4 (10)
	>800	21 (52)
**Position^a^, n (%)**		
	Chief information officer or head of IT	26 (65)
	Chief data officer	1 (3)
	Chief medical officer	1 (3)
	IT department employee	7 (18)
	Research associate	4 (10)
	Data scientist	3 (8)
	No answer	1 (3)
	Other	3 (8)

^a^Selection of multiple items possible.

### Participants’ Professional Opinions and Assessments

Most participants were either undecided or said they rather disagreed with the statement that AI is relevant for the current health care provision in their hospital and in Germany. However, most participants agreed or rather agreed that AI would be relevant in the future, both in Germany (37/40, 93%) and in their own hospital (36/40, 90%). This fits well with most participants fully agreeing or rather agreeing that AI plays a role in their hospital’s strategy (22/40, 55%). On the topic of information about the possible application of AI in hospitals, the participants were more undecided. In all, 13% (5/40) of the participants fully agreed with the statement that they were well informed, and 38% (15/40) of the participants rather agreed that they were well informed. A total of 38% (15/40) of the respondents were undecided, and 13% (5/40) of the respondents said they were rather uninformed. Overall, the participants were rather optimistic about the use of AI technologies in their hospitals. Of the 40 participants, 14 (35%) rather agreed that their hospital was ready for AI, 14 (35%) were undecided, 7 (18%) said they were rather not ready, and only 4 (10%) stated that their hospital was not ready at all. One participant did not respond to this question.

### AI Technologies in Use or in Planning

The next section of the questionnaire focused on AI tools and technologies. In the first subcategory, participants were asked whether their hospital used or planned to use AI technologies. Of the 40 participants, 26 (65%) answered “yes.” Through the following questions, participants were asked to describe these technologies in more detail. Most AI technologies were used or planned for patient care, followed by biomedical research, administration, and logistics and central purchasing. Other areas mentioned by the participants in free text were marketing, malware detection, and pathology. Participants were presented with a list of common AI technologies when they answered “yes” to the first question in this subcategory (Multimedia Appendix 1 provides the full list of technologies). For every listed AI technology, they could categorize their hospital’s current stance on this technology. The options included the following: in planning, in research and developmental stage, implementation phase, routine care, and not applicable. The most commonly chosen technologies overall were as follows: speech recognition and text analysis systems (20/26, 77%, assigned one of the stances other than *not applicable*), systems for picture recognition (17/26, 65%), and robotics and autonomous systems (17/26, 65%).

Sensorics and communication systems were the least picked (10/26, 38%). Most technologies were in the planning phase.

Concerning the integration of these technologies into the overarching system architecture, 27% (7/26) of the participants stated that technologies in their hospital were integrated, in 23% (6/26) of hospitals, technologies were not integrated but integration was planned, 38% (10/26) were partly integrated, and 12% (3/26) were not integrated. In free text, participants provided reasons for the lack of integration, which included missing interfaces; missing standards for interfaces, processes, and organization; unfavorable cost-benefit relationship; missing evaluation and overall concepts; and immaturity of the AI technology.

In a question allowing for multiple choice, participants stated that some or all AI technologies in their institution were commonly developed with industry partners (23/26, 88%) or university-based research partners (9/26, 35%). Only 12% (3/26) of the participants stated that some or all of their AI technologies were developed within their own institutions.

### Barriers to AI Use and Possible Opportunities Associated With AI

The second subcategory included questions about perceived barriers to the use of AI (Table 2). Through a matrix design, we presented the participants with a list of known barriers compiled from the literature. The barrier most participants (36/40, 90%) agreed or partly agreed with was *lacking resources (staff, knowledge, financial)*. Other relevant barriers were *lacking compatibility or interoperability with existing IT infrastructure* (33/40, 83%) and *quality of data* (30/40, 75%). Participants also disagreed or rather disagreed with some of the barriers derived from the literature. Here, the barriers with the least agreement were *leadership acceptance* (4/40, 10%, agreed or rather agreed with the statement) and *patient acceptance* (4/40, 10%). Other barriers with low agreement were *user (eg, physicians and nurses) acceptance* (9/40, 23%) and *corporate culture* (13/40, 33%). In free text, some participants described additional barriers. These contained immaturity of available AI technologies, fear of high expenses in the training and learning phase of AI, and cloud strategies of AI producers.

**Table 2:** Perceived barriers to implementation and use of artificial intelligence (N=40).

Ranking	Barrier	Total participants in agreement and sample percentages, n (%)^a^
1	Lacking resources (staff, knowledge, and financial)	36 (90)
2	Lacking compatibility or interoperability with existing IT infrastructure	33 (83)
3	Quality of data	30 (75)
4	Availability of data	26 (65)
5	Ethical aspects (eg, liability issues)	24 (60)
6	Product range on the market	23 (58)
7	Data protection	22 (55)
7	Quantity of data	22 (55)
8	Legal regulations	19 (48)
9	Consent of the work council	15 (38)
10	Corporate culture	13 (33)
11	User (eg, physicians, nurses, and administration) acceptance	9 (23)
12	Leadership acceptance	4 (10)
12	Patient acceptance	4 (10)

^a^Responses of “agree” or “rather agree” were grouped together.

In the third subcategory, participants were asked about positive prospects possibly associated with AI (Table 3). Then, they had to state their agreement with these opportunities on a 5-point Likert scale. The opportunity with the highest agreement was *increase in efficiency due to time-saving effects* (29/40, 73% agreed or rather agreed with the statement). Other statements also yielded high agreement rates. The opportunity participants agreed with least was *financial savings*. Only 40% (16/40) of the participants said they agreed or rather agreed with the statement that AI could lead to financial savings in their hospital, whereas 40% (16/40) of the participants disagreed or rather disagreed. Overall, this subcategory yielded homogeneous results. No further opportunities were raised in free text.

A detailed presentation and graphs presenting the results of these 2 subcategories can be found in Multimedia Appendix 2.

**Table 3:** Perceived opportunities associated with the implementation and use of artificial intelligence (N=40).

Ranking	Opportunity	Total participants in agreement and sample percentages, n (%)^a^
1	Increase in efficiency due to time-saving effects	29 (73)
2	Competitive advantage	27 (69)
3	Increase in quality of care	25 (66)
4	Easing the workload of employees	21 (53)
5	Financial savings	16 (40)

^a^Responses of “agree” or “rather agree” were grouped together.

### Resources and Requirements for AI Use in Hospitals

For the fourth subcategory, we focused on the resources required for the use of AI technologies in hospitals. Again, the participants were presented with a list of known critical resources for AI implementation, and they had to indicate their level of agreement with these findings from literature (Table 4). The resource most people needed was *staffing resources* (35/40, 90% agreed or rather agreed with the statement). The resource with the least relevance was *organizational frameworks* (25/40, 64%). As seen in the other subcategories, the distribution of answers was homogeneous. A detailed presentation and graphs presenting the results of this subcategory can be found in Multimedia Appendix 2.

**Table 4:** Resources needed for use and implementation of artificial intelligence (N=40).

Ranking	Resource	Total participants in agreement and sample percentages, n (%)^a^
1	Staffing resources	35 (90)
2	Time	34 (87)
3	Knowledge	33 (85)
4	Financial resources	32 (84)
5	Technical resources	31 (79)
6	Data base	27 (69)
7	Organizational frameworks	25 (64)

^a^Responses of “agree” or “rather agree” were grouped together.

The next item asked participants whether their hospital needed to fulfill any further requirements or resources besides those already mentioned in a yes or no format. A total of 60% (24/40) of the participants answered “yes” and provided explanations in free text. Here, organizational aspects were most common (eg, competencies and responsibilities), followed by workflow and legal issues. Technical aspects were described in detail, such as lacking hardware and software, interoperability, difficulties with data transfer from old to new systems, need for additional modules for data capture, and Wi-Fi availability and speed.

Considering the tech industry and its offerings on the market, the participants were highly undecided. Furthermore, 58% (23/40) of the participants said that they did not know if the supply met the demand for AI technologies in their hospital. Only 7% (3/40) of the participants stated that offerings on the market were sufficient.

## Discussion

### Principal Findings

This study provided insights into the current and planned dissemination of AI tools as well as perceived barriers and opportunities for the implementation and adoption of AI tools in 40 hospitals in Germany. We designed a web-based survey based on existing literature on the implementation of AI in hospitals. Our participants were mainly from an IT background, with 28 decision makers in leadership positions. Two-thirds of the participants said that they used or planned to use AI tools in their institution. Speech recognition and text analysis systems, systems for picture recognition, and robotics and autonomous systems were the tools or systems most commonly used, or their use was planned. We did not find differing opinions among hospitals of different sizes or ownership. The results showed that most participants recognized the implementation of AI in hospitals as a relevant, forthcoming part of their IT strategy. However, lack of resources and compatibility or interoperability with the existing IT infrastructure were identified as barriers to implementation. Staffing resources, time, knowledge, financial resources, and technical resources required for the implementation of AI were all highly relevant resources. A possible increase in efficiency because of time-saving effects, competitive advantage, and increase in quality of care was seen as the most important opportunity associated with AI use. We conclude that AI readiness factors derived from the literature are applicable to the hospital context in Germany. The following discussion highlights the most relevant barriers to AI readiness, adoption, and implementation while also presenting possible ways to overcome these barriers.

### AI in Hospital Strategies

AI readiness as a concept has been described recently [15,24]. *Strategic alignment* was identified as 1 of 5 key aspects of organizational AI readiness. Our survey included a question addressing whether AI was a part of the participants’ hospital IT strategy. To this question, 55% (22/40) of the participants agreed or rather agreed that AI was a part of their strategy. In addition, most participants agreed or rather agreed that AI would be relevant in the future, both in Germany (37/40, 93%) and in their own hospital (36/40, 90%). However, this also means that there are decision makers who recognize the relevance of AI in the future but do not consider it a part of their hospitals’ strategy. First, this could be because of the complexity of AI implementation (eg, uncertainties surrounding the workings of the technology, acceptance of the technology, and an unclear regulatory situation) [1,10,17,19,29,35]. Second, the hesitancy to include AI in a hospital’s IT strategy could be explained by high costs and unclear reimbursement schemes [1]. In our study, 80% (32/40) of the participants agreed that their institution lacked financial resources, and 90% (36/40) said that a lack of resources overall was a barrier for AI implementation. At the same time, only 40% (16/40) of the participants agreed with the statement that AI holds a potential for financial savings. This paints a picture of AI as a resource-intensive technology with limited financial rewards. To overcome this barrier and compensate for the financial burden because of investments in digital technologies, the German government recently introduced a new law, the *hospital future act* (ie, Krankenhauszukunftsgesetz). Through this law, hospitals trying to implement digital technologies, including decision support systems, can apply for financial support to facilitate necessary acquisitions [12]. The law went into effect during our data collection period; thus, we cannot report on the possible impacts of this law. However, as the financial aspects were reported as a relevant barrier in our study, it could be of interest for future research to evaluate the effects of the new law.

Although there are both expectations and observations of AI as a possible tool to save cost and generate high revenue [1,6,29], for example, through higher efficiency, high-quality evidence analyzing the cost and benefits of AI implementation in hospitals is missing [7]. Hence, decision makers lack evidence and information, and the business case for AI in hospitals remains unclear [10,29], which in turn inhibits organizational AI readiness [15].

### AI Acceptability

With regard to further barriers to the implementation of AI, *soft* factors such as user, patient, and leadership acceptance were seen as less relevant barriers by the participants in our survey. This impression might be caused by limited contact of IT department members with users, leadership, and especially with patients. Acceptance issues might also become more obvious to decision makers over time, as most participants in our study had not yet implemented AI in their hospital. Nonetheless, it is important to consider the evidence that acceptability is a relevant antecedent of AI adoption and implementation. For example, a paper reviewing 9 studies on the acceptance of AI in health care concluded that consumers have a robust reluctance toward medical care delivered by AI compared with human providers [36]. In another study, only 3% of patients found that the possible negative aspects of AI outweighed the potential benefits [37]. Overall, there is mixed evidence regarding patients’ acceptance of AI in the medical context, and further research is needed [5].

Leadership acceptance and support have been identified as important antecedents for AI implementation [15,24]. The acceptance of AI users, such as physicians and hospital employees, has also been identified as relevant in other studies [9,38]. Following the technology acceptance model, perceived ease of use and usefulness can positively affect favorable attitudes toward a new technology, which in turn improves its acceptability and use [13,39]. Hence, special attention should be paid to these aspects when deciding on acquiring and implementing new AI tools in a hospital.

Finally, the issue of AI acceptability can be addressed by investing in the concept of *explainable AI*, meaning a more transparent, understandable AI with high performance levels [40]. Although little evidence exists, it is reasonable to expect that this new approach could increase AI acceptability by increasing understanding and trust in the new technology [13,40-42]. IT decision makers should not underestimate the issue of AI acceptability and should take the fears and perceptions surrounding AI seriously when planning to implement new AI technology.

### Possible Mismatch in Supply and Demand

Another finding in our study was that only 7% (3/40) of the participants said that the supply of applicable AI solutions to the tech market was sufficient for their needs. Another 58% (23/40) of participants reported that they were unsure. One reason could be that we did not reach the right people in the institution, and they were thus unable to assess the tech market. Another possibility could be that our participants did not spend time researching the offerings in the tech market. This could be especially true for those who are not using or planning to use AI tools. However, it could also be possible that the offerings on the market do not fit the requirements of their potential clients. This result could be of value for tech companies trying to reach decision makers in hospitals. This finding is especially important considering that only 12% of the AI tools were developed within the hospitals in our survey. Hence, partnerships for the development of AI tools are common and must be fostered.

### Generalizability

We created this survey instrument based on an extensive literature research and theoretical frameworks and used cognitive pretesting to ensure understandability. Participants usually completed the survey in <10 minutes. Hence, our survey instrument enabled us to collect data both efficiently and in a theoretically informed manner. This survey could serve as a template for other studies, especially in countries with a similar level of dissemination of AI technologies. Country-specific items, such as the work council, should be adapted to the context in question. Although our survey included these country-specific aspects, they did not appear to be of high relevance in our sample. However, we think that these aspects should be surveyed, as their importance in other contexts is not predictable.

### Strengths and Limitations

This study investigated the status quo of AI technologies in 40 German hospitals and the applicability of AI readiness factors derived from the literature. Owing to the low response rate and resulting small sample size, our results are not representative but describe a first impression. We surveyed hospital CIOs, a group we identified as important intermediaries for digital innovation adoption and implementation. While other studies about the perceptions, barriers, and issues surrounding AI questioned users (eg, physicians and health professionals), patients, or other stakeholders [37,43-45], we focused on the seldom studied group of IT decision makers. Although focusing exclusively on one stakeholder group may introduce a bias, we believe that the focus on this seldom studied group makes our study unique and relevant, thus warranting the risk of bias. The presented perspective of hospital CIOs depicts barriers to AI use and acceptance on a decision or leadership level. Our results can further the holistic discussion about the real-world implementation of AI and AI readiness.

We analyzed the differences in opinions of hospitals differing in size and ownership, which did not produce relevant results. This finding should be interpreted cautiously, as our sample size could be too small to produce significant results.

Owing to technical limitations, we were unable to report the number of unique site visitors. This impedes the calculation of correct survey response rates. Although we used various recruitment methods (emails, letters, and telephone calls) over a prolonged period, our sample size remained small compared with the number of hospitals in Germany (1914 hospitals in 2019 [35]). The small number of respondents may be explained by a general lack of interest in the survey’s topic [46], time constraints because of the COVID-19 pandemic, or because of the requirements of a leadership position and by a hesitancy to click on links sent via email owing to fear of security breaches. We tried to reduce this fear by establishing an *offline* contact with possible participants (letters and telephonic prenotification), but the effect is unclear. At the same time, people who chose to participate in the survey might have a stronger interest or profound experience with AI. We tried to minimize this effect by pointing out in invitations that no knowledge or experience with AI is necessary for participation. However, there was a risk of nonresponse bias in our study.

Considering the demographics of the survey respondents, the sample was very homogeneous, as most participants were middle-aged and male. This distribution was expected and represents the composition of IT departments in Germany [47]. As we included all academic hospitals in our recruitment efforts, larger hospitals were overrepresented in our sample. We expected academic hospitals to be more involved in AI research and thus wanted to invite them to participate in our study. In addition, very small hospitals sometimes do not have a CIO position or outsource their IT services. In such situations, it is possible that our survey invitations did not reach the right person.

As AI is a new and complex technology, it is possible that our participants misunderstood some questions or falsely claimed that they had used AI in their hospital. We managed this risk by closely aligning our survey design with the results from the 6 pretests. Pretest participants suggested not to include a general definition of AI but to give examples for the specific tools in question 2 (“Please assess the current stage of implementation of these AI tools in your hospital”). To keep the survey as short as possible and by keeping in mind that our target group consisted of experts in a related field, we followed this suggestion. However, this risk must be considered when comparing our results.

### Conclusions

This study paints a mixed picture of the status quo of AI in German hospitals. In our sample, few tools have been implemented in routine care, and many hospitals do not use or plan to use AI in the future. This can likely be explained by missing or unclear business cases, which complicates decision-making and resource attribution. We also observed a mismatch or lack of information about AI offerings in the tech market. This is another important aspect to be monitored, as most AI technologies that are already in use were developed in cooperation with external partners. Therefore, these relationships should be fostered. IT decision makers in hospitals should assess their hospitals’ readiness for AI individually with a focus on resources. Further research should continue to monitor the dissemination of AI tools and AI readiness factors to determine whether improvements can be made over time, especially with regard to government-supported investments in AI technologies that could alleviate the financial burden. Qualitative studies with hospital IT decision makers should be conducted to explore the reasons for slow AI adoption in more detail. The results of our study may infer that AI adoption is not only a topic solely for the IT department but also for the whole hospital as an enterprise, including management, medical staff, and business in terms of an important building block of the digital transformation.

## Appendix

Multimedia Appendix 1 Translated survey.

Multimedia Appendix 2 Bar graphs.

## Abbreviations

AI: artificial intelligence
CIO: chief information officer
REDCap: Research Electronic Data Capture
