# Supplementary material for: Perspective of Information Technology Decision Makers on Factors Influencing Adoption and Implementation of Artificial Intelligence Technologies in 40 German Hospitals: Descriptive Analysis
Source: JMIR Med Inform. 2022 Jun 15;10(6):e34678. doi: 10.2196/34678 (PMC9244653; doi:10.2196/34678)
Supplement: Multimedia Appendix 1 [file medinform_v10i6e34678_app1.docx]

**Appendix: Translated survey**

**Survey Title:** *The use of Artificial Intelligence (AI) in German hospitals*

Thank you for your interest in this survey on the topic of “The use of Artificial Intelligence (AI) in German hospitals: a quantitative survey of the status quo, barriers and opportunities of using AI”.

This study is conducted by the Department of Medical Information Systems at the Heidelberg University Hospital and is completely anonymous.

In the following survey, you will be asked for your personal view on different aspects. Please, unless stated otherwise, refer to your personal experiences in your hospital.

You can end the survey at any time and continue later.

This study is funded by the Baden-Wuerttemberg (Germany) Ministry of Science, Research and the Arts under the reference number 42-04HV.MED(19)/15/1 as part of the project ZIV (“Zentrum fuer Innovative Versorgung”). For further information, please contact the principal investigator of this study, Dr. Oliver Heinze (Acting Director of the Department of Medical Information Systems, Heidelberg University Hospital) or the study coordinators Lina Weinert and Julia Müller. Contact data can be found at: www.klinikum.uni-heidelberg.de/mis

1. **General thoughts on AI**

Please assess the following statements:

|  | Fully agree | Partly agree | undecided | Rather disagree | Fully disagree | Don’t know | Prefer not to say |
| --- | --- | --- | --- | --- | --- | --- | --- |
| The use of AI is relevant for the current provision of healthcare in Germany. |  |  |  |  |  |  |  |
| The use of AI is relevant for the future provision of healthcare in Germany. |  |  |  |  |  |  |  |
| The use of AI is relevant for the current provision of healthcare in our hospital. |  |  |  |  |  |  |  |
| The use of AI is relevant for the future provision of healthcare in our hospital. |  |  |  |  |  |  |  |
| The use of AI plays a role in our hospital’s IT strategy. |  |  |  |  |  |  |  |
| I am informed about the possibilities to use AI in hospitals. |  |  |  |  |  |  |  |

1.2 Medical Informatics Initiative

| Do you know about the Federal Ministry of Education and Research’s Medical Informatics Initiative? | Yes | No |
| --- | --- | --- |

If yes:

|  | Fully agree | Partly agree | undecided | Rather disagree | Fully disagree | Don’t know | Prefer not to say |
| --- | --- | --- | --- | --- | --- | --- | --- |
| The Federal Ministry of Education and Research’s Medical Informatics Initiative influences the use of AI in our hospital. |  |  |  |  |  |  |  |

1. **Use of AI tools.**

Examples for AI tools: Systems for picture recognition (e.g. Identification of pathological structures during imaging procedures), systems for Data Management and Analysis (e.g. automated handling of patient flow), assistive systems (e.g. Decision Support Systems for individualized risk prediction), Robotics and autonomous systems (e.g. surgical robots), sensory and communication systems (e.g. automated real-time sensor analysis), speech recognition and text analysis systems (e.g. comparison of literature with patient data via text-mining), virtual reality and augmented reality systems (e.g. two-dimensional, three-dimensional, or four-dimensional visualizations of organs during surgeries).

| Does your hospital use or plan on using AI tools? | Yes | No |
| --- | --- | --- |

If yes:

|  | Patient care | administration | Logistics and procurement | Biomedical research (e.g. laboratories) | other |
| --- | --- | --- | --- | --- | --- |
| Which areas do these tools concern? |  |  |  |  |  |
| Please describe the areas not mentioned in the table above in which your hospital uses or plans on using AI tools. |  | | | | |

Please assess the current stage of implementation of these AI tools in your hospital.

|  | In Planning | Research/Development phase | Implementation | Routine care | Not applicable |
| --- | --- | --- | --- | --- | --- |
| Systems for picture recognition |  |  |  |  |  |
| Systems for Data Management and Analysis |  |  |  |  |  |
| Assistive systems |  |  |  |  |  |
| Robotics and autonomous systems |  |  |  |  |  |
| Sensory and communication systems |  |  |  |  |  |
| Speech recognition and text analysis systems |  |  |  |  |  |
| Virtual reality and augmented reality systems |  |  |  |  |  |
| If applicable: please note AI tools that are in use or in planning in your hospital but were not mentioned above. |  | | | | |

Are AI tools integrated into your hospital’s existing system architecture (usability and interoperability)?

| Yes | Not yet, but in Planning | Partially | No |
| --- | --- | --- | --- |

What is obstructing the full integration into the existing system architecture?

Which external partners were involved in the development of these tools?

| No external partners, developed within the hospital | Non-academic research institutes | academic research institutes | Industry partners | other |
| --- | --- | --- | --- | --- |

Which other partners not mentioned above were involved in the development?

1. **Barriers for AI use**

Please assess the following statement:

I see the following barriers for the use of AI in our hospital:

|  | Fully agree | Partly agree | undecided | Rather disagree | Fully disagree | Don’t know | Prefer not to say |
| --- | --- | --- | --- | --- | --- | --- | --- |
| Data protection/privacy |  |  |  |  |  |  |  |
| Availability of data |  |  |  |  |  |  |  |
| Quality of data |  |  |  |  |  |  |  |
| Quantity of data |  |  |  |  |  |  |  |
| Lacking compatibility/interoperability with existing IT infrastructure |  |  |  |  |  |  |  |
| Legal regulations |  |  |  |  |  |  |  |
| Lacking resources (staff, knowledge, financial, etc.) |  |  |  |  |  |  |  |
| Product range on the market |  |  |  |  |  |  |  |
| Corporate culture |  |  |  |  |  |  |  |
| Leadership acceptance |  |  |  |  |  |  |  |
| User (e.g. doctors, nurses, administration) acceptance |  |  |  |  |  |  |  |
| Patient acceptance |  |  |  |  |  |  |  |
| Consent of the employees’ council |  |  |  |  |  |  |  |
| Ethical aspects (e.g. liability issues) |  |  |  |  |  |  |  |

If applicable, what other barriers not mentioned above do you see for the use of AI tools in your hospital?

1. **Opportunities for AI use**

Please assess the following statement:

I see the following opportunities for the use of AI in our hospital:

|  | Fully agree | Partly agree | undecided | Rather disagree | Fully disagree | Don’t know | Prefer not to say |
| --- | --- | --- | --- | --- | --- | --- | --- |
| Competitive advantage |  |  |  |  |  |  |  |
| Financial savings |  |  |  |  |  |  |  |
| Increase in efficiency due to time saving effects |  |  |  |  |  |  |  |
| Increase in quality of care |  |  |  |  |  |  |  |
| Easing the workload of employees |  |  |  |  |  |  |  |

If applicable, what other opportunities not mentioned above do you see for the use of AI tools in your hospital?

1. **Resources for implementation of AI**

Please assess the following statement:

The following resources are (still) needed for the use of AI tools in our hospital:

|  | Fully agree | Partly agree | undecided | Rather disagree | Fully disagree | Don’t know | Prefer not to say |
| --- | --- | --- | --- | --- | --- | --- | --- |
| Knowledge (e.g. necessary expertise) |  |  |  |  |  |  |  |
| Staffing resources |  |  |  |  |  |  |  |
| Time (e.g. prioritizing AI as a topic) |  |  |  |  |  |  |  |
| Financial resources |  |  |  |  |  |  |  |
| Technical resources (e.g. availability of soft- and hardware) |  |  |  |  |  |  |  |
| Data base |  |  |  |  |  |  |  |
| Organizational frameworks |  |  |  |  |  |  |  |

If applicable, what other resources not mentioned above do you (still) need for using AI tools in your hospital?

Necessary Prerequisites

Does your hospital need to fulfil additional (e.g. technical, organizational) prerequisites for implementing (more) AI tools?

| Yes | No |
| --- | --- |

If yes, what are these?

Offerings on the market

Are there enough offerings on the market to fill the need for AI tools in your hospital?

| Yes | No | I don’t know |
| --- | --- | --- |

1. **Readiness for AI**

Please assess the following statement:

|  | Fully agree | Partly agree | undecided | Rather disagree | Fully disagree | Don’t know | Prefer not to say |
| --- | --- | --- | --- | --- | --- | --- | --- |
| Our hospital is ready for the use of AI tools. |  |  |  |  |  |  |  |

1. **Sociodemographic information**

Please tell us your hospital’s ownership.

| Public | Non-Profit | Private |
| --- | --- | --- |

Please tell us if your hospital is academic or non-academic.

| Academic | Non-Academic |
| --- | --- |

Please tell us what number of beds your hospital has.

1-49, 50-99, 100-149, 150-199, 200-299, 300-399, 400-499, 500-599, 600-799, 800+

Please tell us your position within the hospital. (selection of multiple items possible)

Chief Information Officer/leader of the IT Department

Chief Data Officer

Chief Marketing Officer

IT Department Employee

Research Associate

Data Scientist

No answer

Other:

Please tell us your age.

under 25 years

26 - 35 years

36 - 45 years

46 - 55 years

56 - 65 years

over 65 years

Please tell us your gender.

Male

Female

Diverse

Prefer not to stay

**Thank you for your participation in this survey.** Further information about other projects in the Department of Medical Information Systems, Heidelberg University Hospital, can be found here. (www.klinikum.uni-heidelberg.de/mis)

You may now leave this survey.
