# Supplementary material for: Perspective of Information Technology Decision Makers on Factors Influencing Adoption and Implementation of Artificial Intelligence Technologies in 40 German Hospitals: Descriptive Analysis
Source: JMIR Med Inform. 2022 Jun 15;10(6):e34678. doi: 10.2196/34678 (PMC9244653; doi:10.2196/34678)
Supplement: Multimedia Appendix 2 [file medinform_v10i6e34678_app2.docx]

Fig.1 – Full report on responses to the survey question: “I see the following barriers for the use of AI in our hospital”

Fig. 2 - Full report on responses to the survey question: “I see the following opportunities for the use of AI in our hospital”

Fig. 3 - Full report on responses to the survey question: “The following resources are (still) needed for the use of AI tools in our hospital”
